# Synthesis and Preclinical Evaluation of ^18^F-CB86, a Novel Imidazole[1,2-a] Pyridine-Based TSPO Tracer

**DOI:** 10.3390/ph19101553

**Published:** 2026-10-01

**Authors:** Fan Zhang, Zhanhua Sun, Qiang Zheng, Zhuoxiao Wang, Xilong Tao, Baoqi Huo, Jing Yu, Shisheng Wang

**Affiliations:** 1 Nuclear Medicine Department, The Second Hospital of Dalian Medical University, Dalian 116027, China; zhangfan329@dmu.edu.cn (F.Z.);; 2 State Key Laboratory of Fine Chemicals, School of Pharmaceutical Science and Technology, Dalian University of Technology, Dalian 116024, China

**Keywords:** ^18^F-CB86, TSPO, neuroinflammation, PET

## Abstract

**Background:** The translocator protein (TSPO), an 18 kDa protein localized to the outer mitochondrial membrane, is a recognized biomarker of neuroinflammation. However, its clinical translation has been limited by factors such as poor specificity, low signal-to-noise ratio, and the influence of genetic polymorphisms (rs6971). This study developed a novel TSPO tracer aimed at addressing this technical bottleneck. **Methods**: In this study, a novel TSPO-targeted probe, ^18^F-CB86, was synthesized and labeled using an automated multifunctional module. The radiochemical purity, in vitro stability, and lipid–water partition coefficient of ^18^F-CB86 were systematically evaluated; its biodistribution and Micro-PET/CT imaging characteristics were assessed in a rat model. **Results**: The automated labeling process yielded ^18^F-CB86 with radiochemical purity exceeding 95% and good in vitro stability. Biodistribution studies demonstrated high brain uptake and primary renal clearance. Micro-PET/CT imaging revealed a brain uptake of (SUVmax: 5.91 ± 0.81) % ID/mL at 60 min post-administration, indicating favorable imaging performance. **Conclusions**: These preliminary findings suggest that ^18^F-CB86 is a promising and specific probe for neuroinflammatory imaging and merits further clinical investigation.

## 1. Introduction

The 18 kDa translocator protein (TSPO), also referred to as the peripheral benzodiazepine receptor (PBR), is highly expressed in the mitochondria of human brain microglia, astrocytes, and macrophages [1,2,3,4]. During neuroinflammation, injury, or neurodegenerative disease, microglia are activated, and TSPO expression is markedly upregulated [5]. Multiple studies have demonstrated that PET imaging of TSPO can detect activated microglia in the brain [6,7], establishing that TSPO is a necessary biomarker for in vivo assessment of neuroinflammation. First-generation TSPO tracers, such as ^11^C-PK11195, are limited by low signal-to-noise ratio, high nonspecific binding, and poor sensitivity to low-level inflammation, restricting their clinical utility [8,9,10]. Second-generation tracers, including ^18^F-FEPPA and ^18^F-DPA-714, offer higher affinity [11,12,13,14], but their application is constrained by a common genetic polymorphism (rs6971) in the TSPO, which stratifies individuals into high-, mixed-, and low-affinity binders [11,12,13,14]. This polymorphism significantly influences tracer binding in the brain, complicating data interpretation and necessitating subject genotyping, thereby limiting the broad clinical translation of these tracers [15]. There is an urgent need to develop new TSPO tracers.

CB86 is a ligand with high affinity for TSPO [16,17,18]. Most existing research has focused on radiolabeling CB86 with ^99m^Tc [19]. Analysis of the structure–activity relationship (SAR) and molecular docking of CB86 reveals that the 2-phenyl-3-acetamidoimidazo[1,2-a] pyridine skeleton forms π-π interactions and hydrogen bonds with the aromatic amino acid residues in the TSPO binding pocket, with the pyridine and imidazole nitrogen atoms acting as hydrogen bond acceptors and participating in key polar interactions, thereby facilitating high affinity. Several compounds centered on the imidazo[1,2-a] pyridine core have been reported as TSPO ligands [20,21], indicating that this framework itself exhibits good compatibility with TSPO pharmacophores; simultaneously, multiple sites on this framework are amenable to chemical modification, facilitating the introduction of functional groups such as fluoroacetyl without significantly impairing the binding between the core and the target. The bromoacetyl group can act as a hydrogen-bond donor or acceptor, while the terminal bromine is easily substituted, offering the potential to serve as a linking group for ^18^F radiolabeling. The current study investigates the radiolabeling of CB86 with ^18^F and evaluates its performance in Micro-PET/CT imaging in a rat model of neuroinflammation. PET/CT offers higher image resolution and sensitivity than SPECT, particularly for neuroinflammatory brain imaging, thereby improving the target-to-background ratio. Additionally, small molecules such as ^18^F-CB86 are more lipophilic than metal-coordinating compounds, facilitating blood–brain barrier penetration and enhancing specific TSPO uptake in the brain. The imaging performance and biodistribution patterns were observed for ^18^F-CB86 in a Wistar rat model. Automated modular labeling was used to prepare ^18^F-CB86, and Micro-PET/CT imaging in the Wistar rat model showed that specific uptake of ^18^F-CB86 by TSPO in the hippocampus occurred first; the Micro-PET/CT imaging was conducted to preliminarily assess the probe’s feasibility and specificity for TSPO-targeted neuroinflammatory imaging.

## 2. Results

### 2.1. Synthesis of the Reference Compound ^19^F-CB86

**Figure 1:**
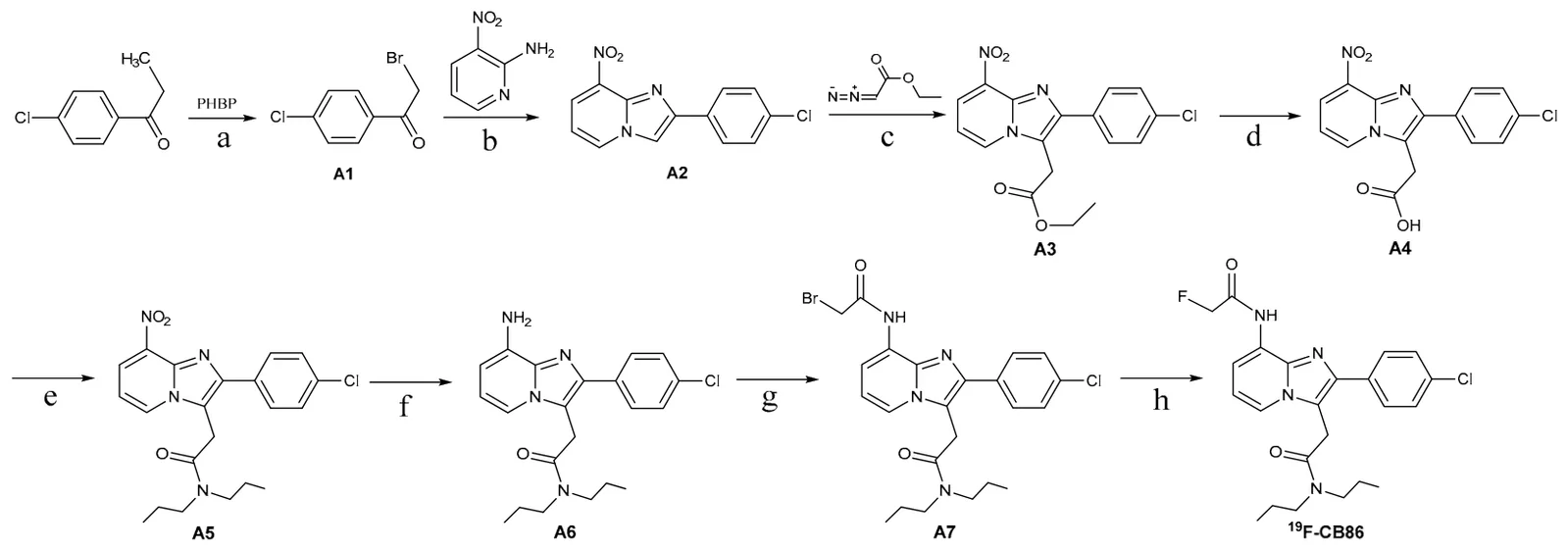
Synthesis route of the reference compound ^19^F-CB86. Reagents and conditions: (a) AcOH, 25 °C, 2 h, 70%; (b) molecular sieve, DMF, N_2_, reflux, 147–150 °C, 6 h, 58%; (c) Cu, toluene, reflux, 120 °C, 2 h, 65%; (d) LiOH, EtOH, reflux, 0–80 °C, 14 h, 81%; (e) n-Dipropylamine, Piv-Cl/K_2_CO_3_, THF, 0 °C, 4 h, 70%; (f) Zn/NH_4_Cl, MeOH, N_2_, 35 °C, 2 h, 86%; (g) bromoacetyl bromide, TEA, DCM, N_2_, 25 °C, 2 h, 80%; (h) KF, diethylene glycol, 150 °C, 2 h, 45%.

All reagents and solvents used in the chemical synthesis were commercially available. The detailed synthetic route is shown in Figure 1; structural identification spectra are displayed in Figures S1–S9. First, chlorophenylacetone was reacted with pyridinium tribromide (PHBP) in glacial acetic acid to yield A1, which was then reacted with 2-amino-3-nitropyridine in DMF to yield A2. Ethyl diazoacetate was dissolved in toluene and, together with active copper powder, added to a toluene solution containing A2 at 120 °C to yield A3. A3 was reacted with lithium hydroxide in ethanol to yield A4, and A4 was reacted with pivaloyl chloride and dipropylamine in tetrahydrofuran to yield A5. A5 was reacted with ammonium chloride and active zinc powder in methanol to yield compound A6 in a high yield (86%). A6 was then immediately dissolved in dichloromethane, and triethylamine and bromoacetyl bromide were added sequentially under mild conditions at 25 °C to yield A7. Finally, A7 was reacted with anhydrous potassium fluoride in a diethylene glycol solution to yield ^19^F-CB86. All intermediates were characterized by ^1^HNMR and MS.

### 2.2. Synthesis and Quality Control of ^18^F-CB86

**Figure 2:**
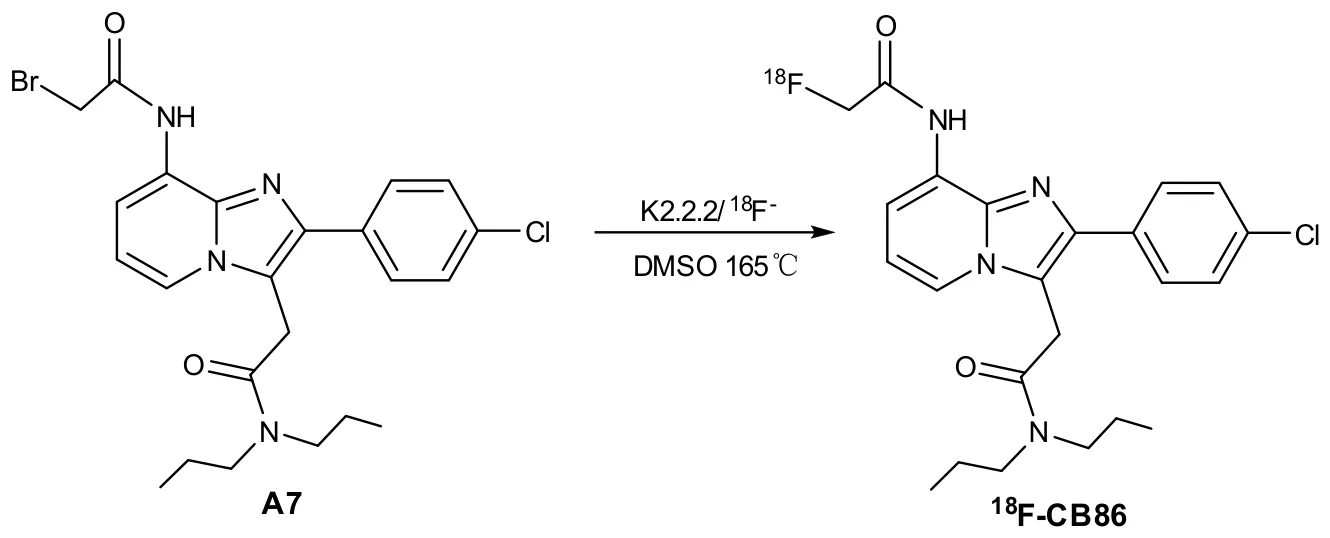
Radioactive labeling of ^18^F-CB86.

**Figure 3:**
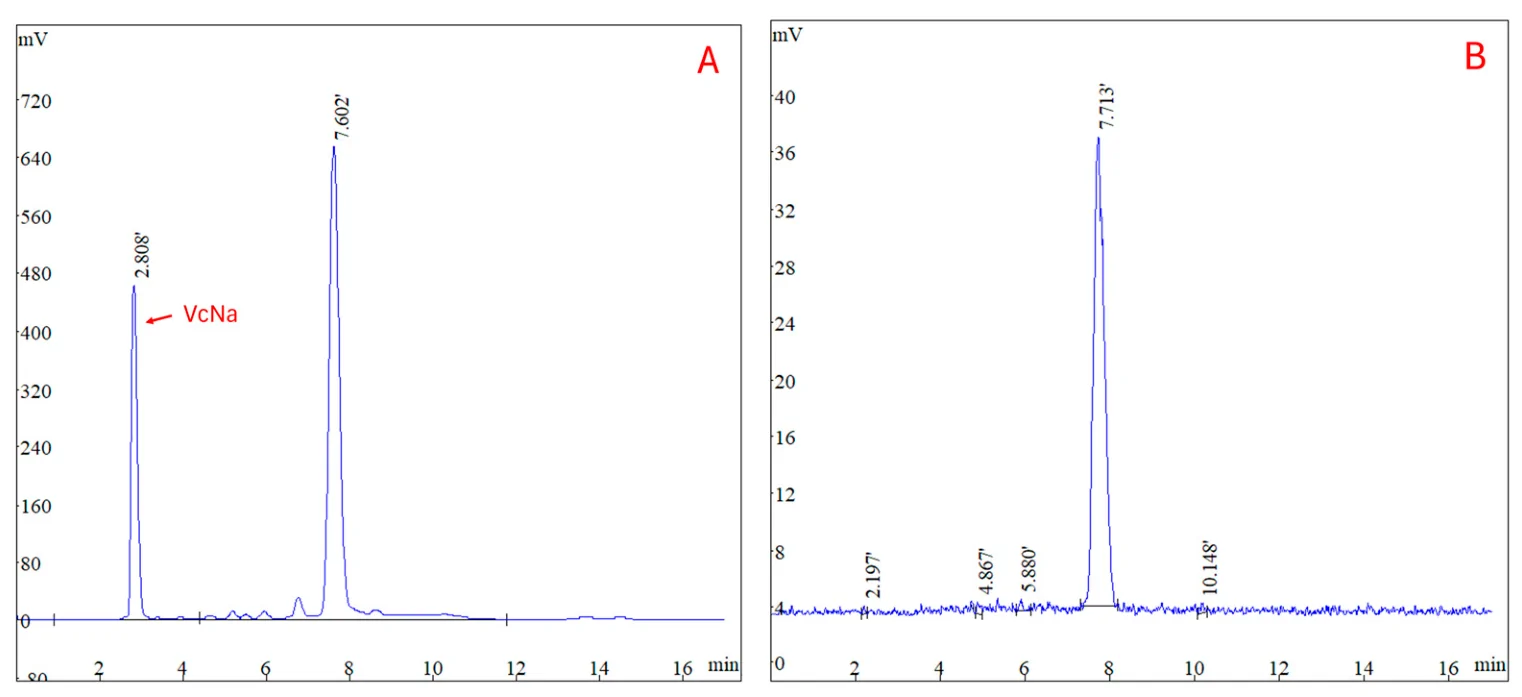
HPLC quality control chart of ^18^F-CB86: (**A**) UV signal, 254 nm; (**B**) radioactivity signal.

^18^F-CB86 was synthesized automatically on a multifunctional synthesis module (Figure 2 and Figure S10). The HPLC quality control results indicate radiochemical purity greater than 95% (Figure 3). ^18^F-CB86 showed a single peak, and the final product was a colorless, clear solution with a pH of approximately 6.0. The UV peak observed at 2.8 min is attributed to the sodium ascorbate (VcNa) added as an antioxidant stabilizer during formulation. Bacterial endotoxin levels were below 15 EU/mL, and ethanol content was 8%. The product met the requirements and is suitable for use in subsequent related studies.

### 2.3. Evaluation of the In Vitro Stability of ^18^F-CB86

**Figure 4:**
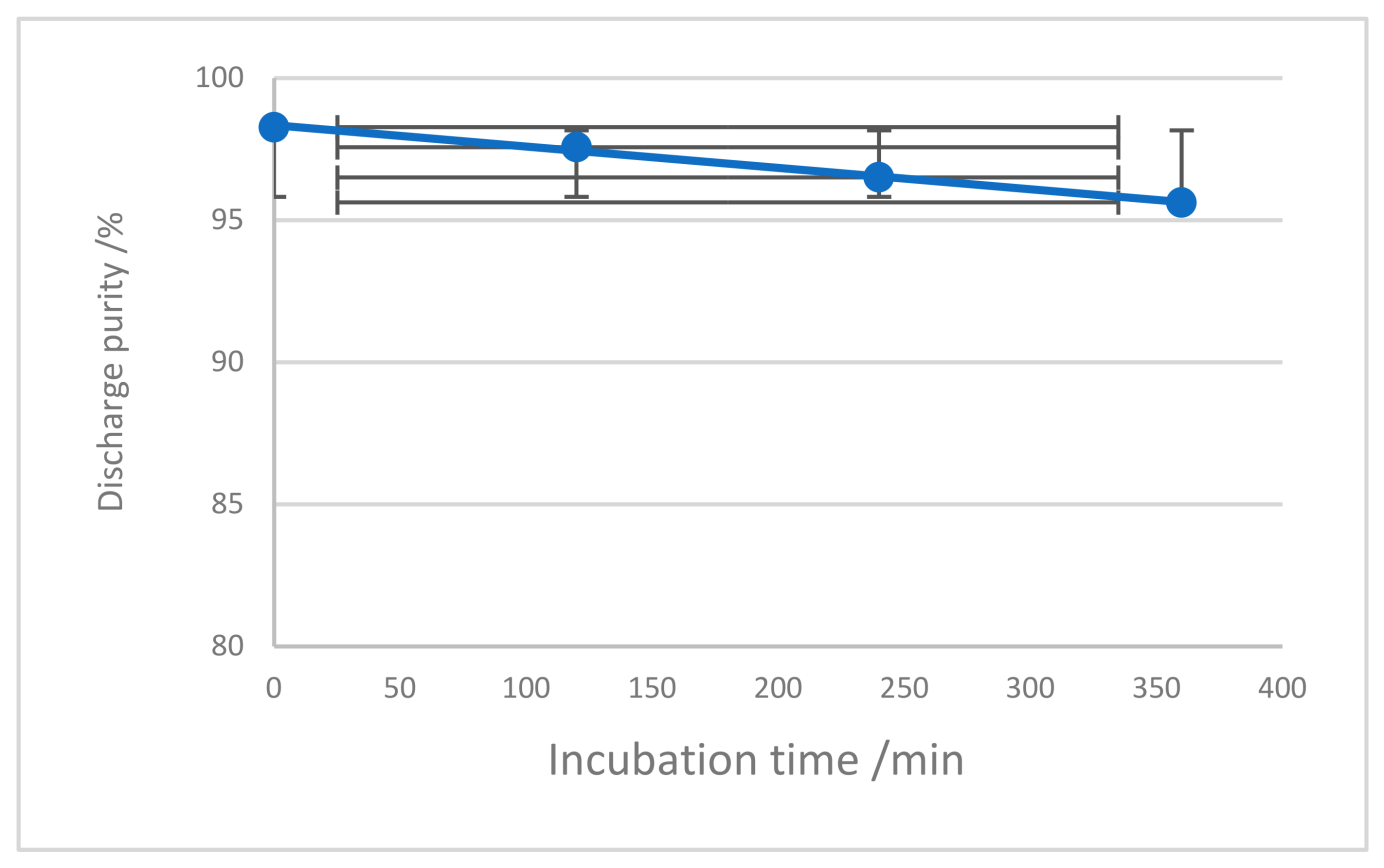
In vitro stability of ^18^F-CB86.

After 6 h of storage at room temperature, the radiochemical purity of ^18^F-CB86 remained above 95% (Figure 4 and Figures S11–S14). Radio-HPLC analysis showed no additional radioactive peaks, indicating that ^18^F-CB86 exhibits good in vitro stability. Lipid–water partition coefficient assays date were listed in Table S1 showed that the log *p* value of ^18^F-CB86 was −0.4624 ± 0.178 (*n* = 3), higher than that of ^99m^Tc-CB86 (log *p* = −1.22 ± 0.04) [22]. This indicates that ^18^F-CB86 is more lipophilic, more readily crosses the blood–brain barrier, and is suitable for neuroinflammatory imaging.

### 2.4. In Vivo Distribution in Rats

**Figure 5:**
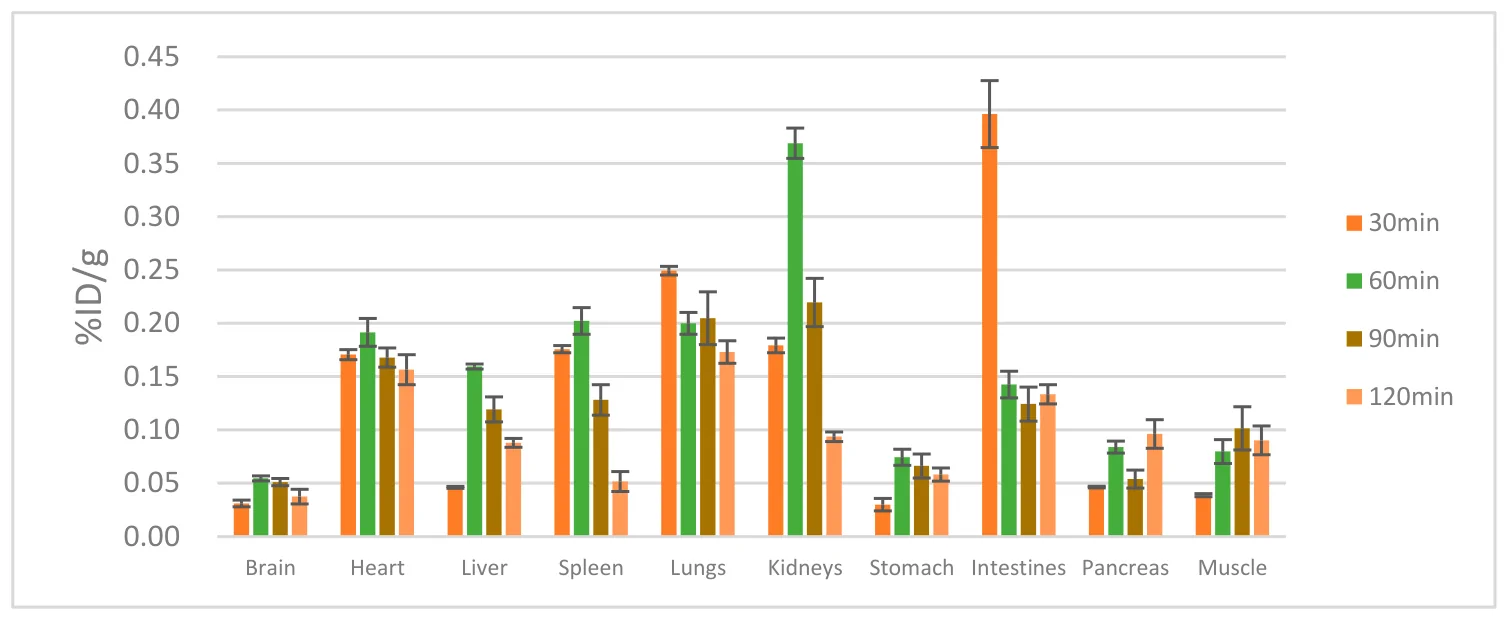
Biodistribution of ^18^F-CB86 in rats.

The in vivo distribution of ^18^F-CB86 in Wistar rats with neuroinflammation is shown in Figure 5 and Table S2. Significant radioactive uptake in the brain was observed 30 min after injection of ^18^F-CB86, peaking approximately 60 min later. It is primarily metabolized via the kidneys. Uptake was also observed in other non-target organs, including the heart, liver, spleen, lungs, and intestines, but rapid metabolism indicates favorable metabolic properties. Statistical comparisons were performed using one-way ANOVA. The results showed that there were significant differences in the %ID/g values across the different time points (*p* < 0.05), and uptake in the 60 min group was significantly higher than that in the 30 min, 90 min, and 120 min groups (*p* < 0.05).

### 2.5. Micro-PET/CT Imaging

**Figure 6:**
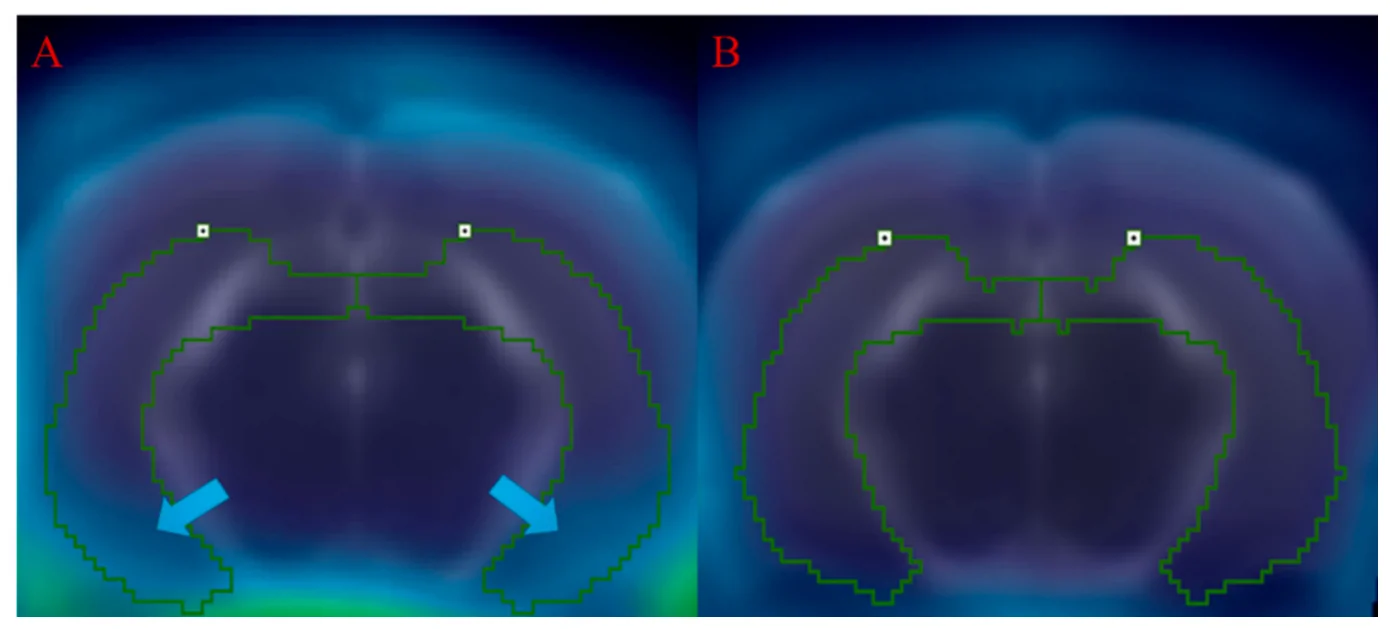
Micro-PET imaging of ^18^F-CB86 in the brain of a rat: (**A**) rat with neuroinflammation; (**B**) control rat.

The Micro-PET imaging results of ^18^F-CB86 in Wistar rats with neuroinflammation are shown in Figure 6, Figures S15 and S16. Sixty minutes after injection,^18^F-CB86 showed significant radioactive accumulation in the hippocampal region of the brain in the neuroinflammatory model rats (SUVmax: 5.91 ± 0.81%ID/mL, SUVmean: 0.67 ± 0.52%ID/mL), whereas uptake in the same region of the control group was significantly reduced (SUVmax: 1.2% ± 0.20%ID/mL, SUVmean: 0.36 ± 0.47%ID/mL).

### 2.6. Adverse Toxicity Study in Rats

The results showed that no rats died within 14 days after injection with ^18^F-CB86 or saline. During the observation period, there were no significant differences between the experimental and control groups in food intake, excreta, activity levels, mental status, or skin condition, and no significant differences in body weight changes (*p* > 0.05). The results are shown in Table 1 and Table S3.

## 3. Discussion

CB86 has been shown to specifically bind to TSPO in diseases such as lung cancer and arthritis [23,24]; however, no reports of its uptake in neuroinflammatory conditions have been published. In this study, we labeled the CB86 ligand with ^18^F to investigate its feasibility as a novel TSPO-targeted molecular probe for neuroinflammatory imaging. Since CB86 has been reported to target TSPO and can serve as an intermediate for molecular probes, this study introduced a fluoroacetyl group at the C-7 position of CB86. However, this modification does not significantly alter affinity, as this region is typically exposed to solvents or lies within the tolerance zone of the binding pocket and does not participate in key interactions. Therefore, the final substituent, the fluoroacetyl group, does not disrupt the parent compound’s key binding mode with TSPO. We have planned to determine the competitive binding affinities (Ki) of CB86 and ^19^F-CB86 for TSPO as a necessary validation step prior to subsequent in vivo experiments. In the chemical synthesis section, we first screened synthetic routes and optimized conditions for the target compound. Drawing on the synthetic protocols for apitan and zopitan, the imidazo[1,2-a] pyridine skeleton was constructed, and the 3-acetamide side chain was introduced via a radical reaction between ethyl diazoacetate and the heterocycle [25,26]. Following further modification of the substituents, the target compound was obtained; simultaneously, the reaction conditions for the skeleton-building step were optimized to improve product yield. In the radiolabeling portion, the entire process was automated, ensuring high reproducibility and reducing the risk of radiation exposure associated with manual operations. The radiochemical purity of the synthesized ^18^F-CB86 exceeded 95%; it is stable in vitro. Micro PET/CT imaging demonstrated radioactive uptake in the hippocampus of rat brains. To avoid potential alterations in biological pathways caused by high tracer doses, this study selected a lower injection dose while ensuring that basic statistical requirements for quantitative analysis were met, which resulted in some limitations on image signal-to-noise ratio and anatomical resolution. To minimize the impact of the low dose on image quality, this study employed an iterative reconstruction algorithm (3D-OSEM) rather than the traditional filtered back-projection (FBP) algorithm for image reconstruction and systematically optimized the reconstruction parameters. Although the low dose resulted in increased image noise and reduced anatomical resolution, the quantitative analyses in this study (such as SUV measurements) were not substantially affected.

However, Micro PET/CT images revealed radioactive uptake in the rats’ bones; the analysis suggests this may be due to in vivo de-labeling. For ^18^F-labeled PET tracers, metabolic defluorination represents a critical liability, as the resulting free ^18^F ion can accumulate in bone and potentially confound quantitative image analysis. Mild skull uptake is observed on the PET images, suggesting low-level defluorination within the body; the potential radioactive metabolites of ^18^F-CB86 remain uncharacterized. Based on the structural features of CB86 (an imidazopyridine acetamide derivative) and the metabolic profiles of related TSPO tracers such as ^18^F-DPA-714, possible metabolic pathways may include oxidative cleavage of the side chain and defluorination at the fluoroethoxy moiety. However, definitive identification requires dedicated metabolite profiling studies. Consequently, readers should interpret the current stability data strictly as evidence of formulation stability rather than biological metabolic stability. Definitive evaluation of the in vivo behavior of ^18^F-CB86—including parent fraction over time, identification of major radiometabolites, quantitative assessment of defluorination, and correlation with in vitro observations—requires systematic plasma metabolite analysis using radio-HPLC coupled with mass spectrometric identification. We have identified this as a critical direction for our subsequent preclinical pharmacokinetic studies. Since this study provides only preliminary insights into the tracer’s in vitro stability, the culture medium used cannot fully simulate the in vivo enzymatic degradation environment, and in vivo metabolism is influenced by additional factors. Due to limitations in the experimental timeline and the radiolabeling schedule, we have listed these as necessary follow-up experiments. We plan to supplement the stability assessment in rat serum and plasma, complete the metabolite analysis of brain tissue, and promptly incorporate the data upon acquisition. We will then analyze the results to guide further research on the structure–activity relationship of the drug, optimize the molecular structure, and improve the tracer-to-target ratio.

Since TSPO levels in the hippocampus of neuroinflammatory rats are significantly elevated compared to those in normal rats [27], to evaluate the binding capacity of the ^18^F-CB86 probe to the TSPO target in the brain and its biodistribution in vivo, 24 healthy adult male Wistar rats were selected. A neuroinflammatory rat model was established using 0.05% (*w*/*v*) propylthiouracil (PTU). Since neuroinflammation and TSPO expression exhibit significant sexual dimorphism and age-related differences, future studies will be conducted in female rats and aged-animal models, and the effects of genetic background on tracer metabolism, target binding, and off-target signaling will be evaluated in at least one additional strain or species. Furthermore, the PTU-induced neuroinflammatory model primarily activates glial cells and elevates TSPO expression indirectly through hypothyroidism; its advantages lie in its stability, reproducibility, and ease of use, making it suitable as a preliminary screening tool for TSPO tracers. However, the pattern of TSPO elevation in this model does not fully reflect the state of glial cell activation observed in chronic neurodegenerative diseases, nor is it entirely equivalent to the pathological processes in primary neuroinflammatory diseases such as Alzheimer’s disease, Parkinson’s disease, or multiple sclerosis. This study used the PTU model for preliminary screening to rapidly assess the uptake and specificity of CB86 in brain regions with elevated TSPO levels, rather than as an authoritative representation of human neuroinflammatory pathology. Before proceeding to clinical trials, CB86 must be validated in at least one other model (such as an LPS-induced acute neuroinflammatory model or a transgenic model with stronger disease relevance). Furthermore, a comprehensive evaluation must be conducted by integrating data on TSPO expression kinetics, tracer metabolism, and specific binding from different inflammatory models. The preliminary results of 18F-CB86 demonstrate potential value in human neuroinflammatory imaging and suggest possible future applications in patients with neurodegenerative diseases or acute inflammation; however, issues such as dosimetry, specificity, and safety, which remain to be resolved in the transition from animal models to human studies, must be addressed in subsequent research.

Comparison with Existing Tracers: ^18^F-DPA-714 is a second-generation TSPO ligand. In TSPO binding assays on rat renal cell membranes, the dissociation constant (Ki) of DPA-714 was 7.0 ± 0.4 nM [28], while in vitro binding assays with CB86 showed a TSPO affinity (Ki) of 1.6 nM [29]. This affinity value is approximately four times that of DPA-714, suggesting that CB86 binds more tightly to TSPO at the molecular level. DPA-714 exhibits good blood–brain barrier permeability. In rodents, ^18^F-DPA-714 uptake in multiple brain regions exceeds 3% ID/mL [30], and it has a long imaging window of 20–40 min after injection, making it suitable for longitudinal studies of neuroinflammation. In this study, dynamic PET scans showed that ^18^F-CB86 accumulated in the rat brain, reaching a peak at 60 min with a maximum uptake rate of up to 9% ID/mg, followed by slow clearance. This performance was superior to that of ^18^F-DPA-714, demonstrating the imaging advantages of CB86. Due to insufficient data on pharmacokinetics and specific binding, we plan to conduct these experiments in future research to provide additional data support for the preclinical studies of ^18^F-CB86.

Furthermore, because existing tracers are affected by the rs6971 polymorphism, there is a significant difference in signal intensity between high-affinity and low-affinity binders, which limits the universality of quantitative analysis. The theoretical advantage of CB86 lies in the fact that, based on the chemical structure of the CB86 ligand, we hypothesize that its binding mode may not depend on the key amino acid residues involved in the rs6971 polymorphism, thereby potentially maintaining relatively consistent affinity across different genotypes. Furthermore, the unique lipophilicity and metabolic characteristics of CB86 may reduce the uptake of radioactive metabolites by the brain and enhance signal specificity. These advantages are currently inferred from structure–activity relationships and need to be confirmed by directly measuring binding affinity in cells or tissues expressing different TSPO genotypes; relevant experiments have been scheduled as part of future work.

This study has several limitations. According to the literature, immunohistochemistry (IHC) and Western blot results have confirmed the upregulation of TSPO in the model [27], but we have not yet conducted in vivo blockade experiments using validated TSPO ligands (such as PK11195, FEPPA, or DPA-714), nor have we performed in vitro competitive binding assays to quantitatively determine the binding affinity of this tracer. These experiments are crucial for comprehensively validating TSPO-specific binding and are planned as part of our ongoing follow-up research. Additionally, we lack a direct quantitative correlation analysis at the individual level between ^18^F-CB86 PET regional uptake and in vitro TSPO expression. Although this is the gold standard for the biological validation of TSPO tracers, we are currently unable to conduct this analysis due to limitations in the availability of paired biological samples. However, the consistency at the population level between regions of high PET uptake and enhanced TSPO immunohistochemical positivity, combined with extensive published evidence from similar TSPO ligands, collectively supports the target specificity of in vivo ^18^F-CB86 uptake. We plan to formally conduct a combined analysis of quantitative PET and proteomics in subsequent translational studies.

Future research directions include integrating artificial intelligence through automated algorithms to optimize the molecular structure of the CB86 ligand to enhance specificity, and exploring conjugation with other radionuclides (such as ^177^Lu and ^225^Ac) to achieve an integrated “diagnosis-treatment” approach.

## 4. Materials and Methods

### 4.1. Experimental Materials

#### 4.1.1. Major Instruments

Sumitomo HM-10S Cyclotron: Sumitomo (Tokyo, Japan); PET-MF-2V-IT-I Multifunctional Synthesis Module: Paite (Beijing, China) Technology Co., Ltd.; Endotoxin Rapid Test System (PTS): Charles River Laboratories (Zhanjiang Andus Biotechnology Co., Ltd., Zhanjiang, China); Preparative High-Performance Liquid Chromatography (HPLC) System (Alltech SS420-X Workstation, equipped with a 515 pump and FR-1000 flow-through radioactivity detector): Paite (Beijing, China) Technology Co., Ltd.; Analytical Column: Phenomenex Gemini 5μ100 A C18 (4.6 mm × 150 mm); LC-16Analytical High-Performance Liquid Chromatography (HPLC) System: Shimadzu Corporation, Kyoto, Japan; ZDN-2 NA-I γ-ray Energy-Dispersive Spectroscopy System: Beijing Zhongzhihean Technology Co., Ltd., Beijing, China; Micro PET/CT System: PingSheng Medical Technology (Kunshan) Co., Ltd., Beijing, China; SLGVR33RS sterile liquid filter membrane: Millipore Corporation, Burlington, MA, USA; CRC-25R activity meter: Capintec, Florham Park, NJ, USA; Bruker Avance II 400 or Vaian DLG 400 Nuclear Magnetic Resonance Spectrometer: Varian, Palo Alto, CA, USA; TSQ Quantum Ultra Triple Quadrupole Liquid Chromatography–Mass Spectrometry System: Thermo Fisher Scientific, Inc., St. Louis, MO, USA.

#### 4.1.2. Experimental Animals

A total of 24 healthy adult male Wistar rats (approximately 8 weeks old) were used in this study. Rats were housed two per cage and maintained at 22 ± 1 °C and 50–60% humidity, with a regular light–dark cycle (lights on at 8:00 a.m. and off at 8:00 p.m.). All rats were randomly assigned to two groups: the neuroinflammatory group and the blank control group (hereinafter, the control group). Body weight was measured and recorded weekly for all rats. During the modeling period, propylthiouracil (PTU) was dissolved in drinking water to prepare a 0.05% (*w*/*v*) PTU solution, which was made freely available to rats in the neuroinflammatory group. Rats in the control group had access to an unlimited supply of drinking water. The entire modeling process lasted 8 weeks. All rats were fed the same amount of laboratory chow. Before the experiment, all animals were acclimated to the experimental environment for at least one week. The PTU-induced rat model is widely used in neuroinflammation research because PTU administration reliably induces hypothyroidism-associated neuroinflammatory changes and upregulates TSPO expression, making it well-suited for evaluating TSPO-targeted imaging probes. This study was conducted in accordance with the Chinese “Guidelines for the Ethical Review of Laboratory Animal Welfare,” and the Animal Research Committee of Dalian Medical University approved all animal procedures.

### 4.2. Experimental Methods

#### 4.2.1. Synthesis of 2-Bromo-4′-chloroacetophenone (A1)

First, 1 g of p-chloroacetophenone (6.49 mmol) was dissolved in 30 mL of ice-cold acetic acid. Then, 3.11 g of pyridinium tribromide (PHBP) (9.74 mmol) was added and the mixture stirred at room temperature. Monitoring was conducted using TLC; the reaction was complete after 2 h. The reaction mixture was left open overnight in a fume hood, then centrifuged to remove the supernatant. The residue was purified by silica gel column chromatography using petroleum ether–ethyl acetate (5:1, *v*/*v*). 2-Bromo-4′-chlorophenylacetone (A1, white crystals, 1.05 g, 70% yield) was obtained. ^1^H NMR (400 MHz, CDCl_3_) δ 7.93 (d, *J* = 8.6 Hz, 2H), 7.48 (d, *J* = 8.6 Hz, 2H), 4.41 (s, 2H).

#### 4.2.2. Synthesis of 2-(4-Chlorophenyl)-8-nitroimidazo[1,2-a] Pyridine (A2)

First, 1 g of compound A1 (4.28 mmol) and 0.596 g of 2-amino-3-nitropyridine (4.28 mmol) were dissolved in 3.5 mL of anhydrous DMF, 4A zeolite was added, and the reaction was refluxed under a nitrogen atmosphere while monitored by TLC; the reaction was complete after 6 h. The reaction mixture was filtered through diatomaceous earth, then centrifuged to dryness, and finally purified by column chromatography on silica gel (petroleum ether–ethyl acetate, *v*/*v* = 3:1–1:1). 2-(4-chlorophenyl)-8-nitroimidazo[1,2-a] pyridine (A2, yellow crystals, 0.68 g, 58% yield) was obtained. ^1^H NMR (400 MHz, CDCl_3_) δ 8.43 (d, J = 6.5 Hz, 1H), 8.18 (d, *J* = 7.6 Hz, 1H), 8.03 (s, 1H), 7.96 (d, *J* = 8.4 Hz, 2H), 7.42 (d, *J* = 8.4 Hz, 2H), 6.94 (t, *J* = 7.1 Hz, 1H).

#### 4.2.3. Synthesis of Ethyl 2-(2-(4-Chlorophenyl)-8-nitroimidazo[1,2-a] Pyridin-3-yl) Acetate (A3)

First, 1.05 mL of ethyl diazoacetate (9.91 mmol) was dissolved in 1 mL of toluene, then added to a 30 mL solution of 2.26 g of compound A2 (8.26 mmol) in anhydrous toluene at 120 °C. The active copper powder (0.431 g, 6.77 mmol, activated with 1 mol/L HCl for 30 min, washed successively with water, methanol, and THF, and dried under vacuum for 7 h) was added at 120 °C. The diazoacetic acid ethyl ester and copper powder were added in five portions. The resulting slurry was heated at 120 °C and cooled to room temperature after 2 h. The mixture was filtered and concentrated. The viscous residue was used as the starting material, and the reaction was repeated three times using the method described above. The crude product was recrystallized from petroleum ether–ethyl acetate (1:1, *v*/*v*). Ethyl 2-(2-(4-chlorophenyl)-8-nitroimidazo[1,2-a] pyridin-3-yl) acetate (A3, pale yellow solid, 1.93 g, 65% yield) was obtained. ^1^H NMR (400 MHz, CDCl_3_) δ 8.50 (d, *J* = 6.5 Hz, 1H), 8.25 (d, *J* = 7.6 Hz, 1H), 7.84 (d, *J* = 8.3 Hz, 2H), 7.48 (d, *J* = 8.2 Hz, 2H), 7.05 (t, *J* = 7.1 Hz, 1H), 4.25 (q, *J* = 7.1 Hz, 2H), 4.08 (s, 2H), 1.29 (t, *J* = 7.1 Hz, 3H).

#### 4.2.4. Synthesis of 2-(2-(4-Chlorophenyl)-8-nitroimidazo[1,2-a] Pyridin-3-yl) Acetic Acid (A4)

First, 0.78 g of compound A3 (2.17 mmol) was dissolved in 50 mL of anhydrous ethanol and cooled to 0 °C. Then, 6.3 mL of an aqueous lithium hydroxide solution (6.3 mmol, 1 mol/L) was added dropwise, then slowly heated to reflux. The mixture was left to react for 14 h, and monitored via TLC until completion. The ethanol was evaporated under vacuum, 50 mL of water was added, and the reaction mixture was extracted with ethyl acetate (2 × 20 mL). The collected aqueous layer was adjusted to pH 3 with 1 mol/L HCl (7 mL) at 0 °C, then extraction was performed with dichloromethane (3 × 30 mL). The combined organic layers were dried over anhydrous Na_2_SO_4_. The Na_2_SO_4_ was filtered off and the filtrate concentrated to dryness. 2-(2-(4-chlorophenyl)-8-nitroimidazo[1,2-a] pyridin-3-yl) acetic acid (A4, brown solid, 0.58 g, 81% yield) was obtained. ^1^H NMR (400 MHz, CD_3_OD) δ 8.78 (d, *J* = 6.8 Hz, 1H), 8.40 (d, *J* = 8.3 Hz, 1H), 7.80 (d, *J* = 8.6 Hz, 2H), 7.55 (d, *J* = 8.5 Hz, 2H), 7.25–7.16 (m, 1H), 4.18 (d, *J* = 7.1 Hz, 2H).

#### 4.2.5. Synthesis of 2-(2-(4-Chlorophenyl)-8-nitroimidazo[1,2-a] Pyridin-3-yl)-N, N-dipropyl Acetamide (A5)

First, 0.26 g of compound A4 (0.772 mmol) and 118 mg of potassium carbonate (0.85 mmol) were added to 10 mL of anhydrous tetrahydrofuran. The reaction mixture was cooled to 0 °C, followed by the addition of 0.11 mL of pivaloyl chloride (0.92 mmol) and 0.16 mL of dipropylamine (1.12 mmol). The reaction was monitored via TLC; it was complete after 4 h. A total of 15 mL of water was added, and the mixture was extracted with ethyl acetate (3 × 10 mL). The combined ethyl acetate organic layer was washed with 15 mL of saline solution, then dried over anhydrous Na_2_SO_4_. The Na_2_SO_4_ was filtered off and the filtrate concentrated to dryness. The crude product was purified by rapid column chromatography on silica gel. 2-(2-(4-chlorophenyl)-8-nitroimidazo[1,2-a] pyridin-3-yl)-N, N-dipropyl acetamide (A5, pale yellow solid, 0.22 g, 70% yield) was obtained. ^1^H NMR (400 MHz, CDCl_3_) δ 8.65 (d, *J* = 6.8 Hz, 1H), 8.22 (d, *J* = 7.5 Hz, 1H), 7.65 (d, *J* = 8.4 Hz, 2H), 7.46 (d, *J* = 8.4 Hz, 2H), 6.97 (t, *J* = 7.2 Hz, 1H), 4.14 (d, *J* = 6.3 Hz, 2H), 3.33–3.21 (m, 2H), 3.13–3.03 (m, 2H), 1.51 (m, *J* = 11.2, 7.5 Hz, 4H), 0.84 (t, *J* = 7.4 Hz, 3H), 0.74 (t, *J* = 7.4 Hz, 3H).

#### 4.2.6. Synthesis of 2-(8-Amino-2-(4-chlorophenyl) imidazole[1,2-a] Pyridin-3-yl)-N, N-dipropyl Acetamide (A6)

First, 0.17 g of compound A5 (0.4 mmol) was dissolved in 30 mL of methanol (90%, *v*/*v*). A total of 0.19 g of ammonium chloride (0.9 mmol) was added, followed by the addition of 1 g of active zinc powder (15.3 mmol) at 37 °C. The mixture was stirred for 2 h, and then the solid was filtered through a diatomaceous earth pad. The filtrate was concentrated, followed by the addition of 20 mL of ethyl acetate and 10 mL of water. The organic layer was extracted with ethyl acetate (3 × 5 mL). The organic layers were combined, washed with 10 mL of saline solution, dried over anhydrous Na_2_SO_4_, filtered, and concentrated to dryness. The crude product was purified by rapid silica gel chromatography using dichloromethane–ethyl acetate (10:1, *v*/*v*). Compound A6 was obtained as a white solid (0.13 g, 86% yield) and was used immediately in the next step of the synthesis.

#### 4.2.7. Synthesis of 2-(8-(2-Bromoacetamido)-2-(4-chlorophenyl) imidazo[1,2-a] Pyridin-3-yl)-N, N-dipropylacetamide (A7)

To a stirring solution of 0.2 g of compound A6 (0.52 mmol) in anhydrous dichloromethane, 0.079 mL of triethylamine (0.57 mmol) was added, followed by the addition of 0.05 mL of bromoacetic acid bromide (0.57 mmol). The mixture was stirred for 2 h at 25 °C under a nitrogen atmosphere. The solvent was removed under reduced pressure, and then the solid residue was treated with 25 mL of 10% NaHCO_3_ solution. The mixture was extracted with dichloromethane (3 × 20 mL). After drying with Na_2_SO_4_, the solvent was removed to obtain a solid residue, which was purified by silica gel chromatography using petroleum ether and ethyl acetate (7:3, *v*/*v*) as the eluent. 2-(8-(2-bromoacetamido)-2-(4-chlorophenyl) imidazo[1,2-a] pyridin-3-yl)-N, N-dipropyl acetamide (A7, brownish-yellow solid, 0.21 g, 80% yield) was obtained. ^1^H NMR (400 MHz, CDCl_3_) δ 9.66 (s, 1H), 8.21 (d, *J* = 7.5 Hz, 1H), 7.95 (d, *J* = 6.9 Hz, 1H), 7.63 (d, *J* = 8.5 Hz, 2H), 7.45 (d, *J* = 8.5 Hz, 2H), 6.88 (t, *J* = 7.2 Hz, 1H), 4.11 (s, 2H), 3.33–3.24 (m, 2H), 3.17–3.08 (m, 2H), 1.56–1.47 (m, 4H), 0.85 (s, 3H), 0.76 (t, *J* = 7.4 Hz, 3H). ESI-MS Calcd. for C_23_H_26_BrClN_4_O_2_ [M + Na]^+^ 528.84, found: 528.96.

#### 4.2.8. Synthesis of 2-(8-(2-Fluoroacetamido)-2-(4-chlorophenyl) imidazo[1,2-a] Pyridin-3-yl)-N, N-dipropylacetamide (^18^F-CB86)

First, 1.77 g of compound A7 (3.5 mmol) and 5.2 g of anhydrous potassium fluoride (8.9 mmol) were dissolved in 5 mL of diethylene glycol, stirred, rapidly heated to 150 °C, and held at that temperature for 2 h. The reaction mixture was cooled, diluted with 10 mL of water, and extracted with toluene (3 × 10 mL). The organic layer was washed with 10 mL of 5% sodium bicarbonate solution, dried over MgSO_4_, filtered, and concentrated. 2-(8-(2-fluoroacetamido)-2-(4-chlorophenyl) imidazo[1,2-a] pyridin-3-yl)-N, N-dipropyl acetamide (^19^F-CB86, white solid, 0.71 g, 45% yield) was obtained. ^1^H NMR (400 MHz, CDCl3) δ 8.46 (s, 1H), 8.00 (d, *J* = 6.7 Hz, 1H), 7.66 (d, *J* = 8.5 Hz, 2H), 7.49 (d, *J* = 8.4 Hz, 2H), 7.05 (s, 1H), 4.09 (s, 2H), 3.34–3.29 (m, 2H), 3.20–3.15 (m, 2H), 1.56–1.60 (m, 4H), 0.87–0.76 (m, 6H). ESI-MS Calcd. for C_23_H_26_ClFN_4_O_2_ [M + H]^+^ 445.17, found: 445.14. Corresponding 1H NMR data are reported in reference [16].

### 4.3. Preparation of ^18^F-CB86

Radioactive labeling was performed using the multifunctional synthesis module and a Sumitomo HM-10 cyclotron set to a beam current of 60 μA. The ^18^F ion was produced via the ^18^O (p, n)^18^F reaction, yielding 823 mCi. Helium was used to transfer the ^18^F ion from the cyclotron to the QMA column of the module. The ^18^F ion was eluted into a reaction tube using 1.5 mL of a K2.2.2/K_2_CO_3_ solution (containing 4.15 mg of K2.2.2 and 1.04 mg of K_2_CO_3_). The reaction tube was heated to 116 °C, and nitrogen was introduced until the liquid had completely evaporated. Then, 2 mL of acetonitrile was added, and the mixture was heated to 116 °C under nitrogen until the liquid had completely evaporated, followed by continued heating for 30 s. Next, 1 mL of an 8 mg/mL DMSO solution of the CB86 precursor was added. The mixture was heated to 165 °C to achieve a nucleophilic reaction for 20 min, then cooled to 45 °C, diluted with 4 mL of a 50% ethanol–water solution, transferred to a transfer flask, and purified via preparative liquid chromatography. The mobile phase consisted of 50% ethanol–water solution (containing 0.2% H_3_PO_4_ by volume) at a flow rate of 5 mL/min. The radioactive product peak was collected at 7.1 min into a transfer flask, and filtered through a 0.22 μm sterile filter membrane to obtain ^18^F-CB86 injection; the final formulation contained sodium ascorbate (VcNa, 2 g/L). The decay-corrected radiochemical yield (RCY) was 12.9%, with radiochemical purity (RCP) of 97.9% and molar activity (MA) of 154 GBq/μmol. The total synthesis time of the probes was 32 min.

### 4.4. Quality Control of ^18^F-CB86

The product’s color and clarity were observed through lead glass. pH was measured with precision pH paper; radiochemical purity was measured with an analytical Radio-HPLC system using a 50% (*v*/*v*) ethanol–water mobile phase; and endotoxin content was measured with a portable endotoxin detector.

### 4.5. In Vitro Stability

The collected ^18^F-CB86 injection solution (approximately 52 MBq activity; radioactivity concentration of 103 MBq/mL) was incubated at room temperature for 6 h. Samples were collected at 0, 2, 4, and 6 h, respectively, and radiochemical purity was assessed by radio-HPLC.

### 4.6. Lipid–Water Partition Coefficient

First, 100 μL of ^18^F-CB86 solution (approximately 20 μCi), 1.5 mL of n-octanol, and 1.5 mL of PBS (0.1 mol/L, pH = 7.4) were added to the tube. Each tube was vortexed thoroughly for 3 min at room temperature, centrifuged at 4000 rpm for 5 min, then allowed to stand until stratified. A total of 250 μL of each of the upper n-octanol layer and the lower PBS layer was transferred into counting tubes. This procedure was performed in triplicate, and the radioactivity counts (cps) of each sample were measured using a γ-spectrometer. Log *p* was calculated as Log (counts in n-octanol/counts in PBS). The experimental procedure was repeated three times.

### 4.7. In Vivo Distribution in Rats

Twelve male Wistar rats (approximately 8 weeks old) with neuroinflammation were selected. Each rat received 0.2 mL of ^18^F-CB86 (approximately 37 MBq) via the tail vein. Rats were euthanized at 30, 60, 90, and 120 min post-injection. Organs and tissues, including the brain, heart, liver, spleen, lungs, kidneys, stomach, intestines, pancreas, and muscle, were harvested, cleaned, and weighed, and their radioactivity was measured with a γ-spectrometer. Three rats were used per time point. The percentage of the injected dose per gram (% ID/g) was calculated for each tissue. %ID/g was used for in vivo gamma counter data, and SUV was used for in vivo PET image analysis, along with their mathematical relationship: %ID/g = SUV × 100/body weight (g). Data are presented as mean ± standard deviation (SD). Statistical analysis was performed using one-way analysis of variance (ANOVA) to compare uptake across time points; *p* < 0.05 was considered statistically significant.

### 4.8. Micro-PET/CT Imaging in Rats

Male Wistar rats (approximately 8 weeks old) with neuritis were administered 0.2 mL of ^18^F-CB86-labeled compound (approximately 37 MBq) via the tail vein, and Micro-PET imaging was performed 60 min after injection. To immobilize the animals, imaging was performed under isoflurane anesthesia (5% induction, 2–2.5% maintenance), with the head secured with tape to prevent unintended movement during the scan. The imaging session lasted 20 min. After image acquisition, images were reconstructed using the three-dimensional ordered-subset maximum-likelihood (3D-OSEM) algorithm, and the hippocampus was analyzed as the region of interest in Pmod 4.304 software.

### 4.9. In Vivo Toxicity Study in Rats

Twelve male Wistar rats, approximately 8 weeks old and weighing 421–447 g, were selected and randomly assigned to an experimental group and a control group (n = 6). The body weights of the rats in each group were measured and recorded before the experiment.

## 5. Conclusions

A novel imidazo[1,2-a] pyridine-based TSPO tracer, ^18^F-CB86, was synthesized and labeled using an automated multifunctional module. The target compound demonstrated high radiochemical purity and stability. In vivo distribution studies in rats with neuroinflammation showed high brain uptake and primary renal metabolism of ^18^F-CB86. Micro-PET imaging produced high-quality hippocampal images, supporting the probe’s feasibility and specificity for neuroinflammatory imaging. Further evaluation and potential structural modifications are recommended to optimize performance. Preclinical animal studies indicate the potential of ^18^F-CB86 as a PET molecular probe for TSPO. Future research will include direct comparative studies of ^18^F-CB86 with established TSPO tracers such as ^11^C-PK11195, ^18^F-FEPPA, and ^18^F-DPA-714 to assess its imaging characteristics and clinical potential more comprehensively. Additionally, structural modifications will be explored to enhance blood–brain barrier penetration and binding affinity further, and the tracer will be evaluated in larger animal models before clinical translation.

## Figures and Tables

**Table 1:** Body weight changes in rats injected with ^18^F-CB86 or saline (*n* = 6).

	Experimental Group			Control Group			
Radiopharmaceutical	Body weight before administration/g	Body weight 14 days after administration/g	Weight gain/g	Body weight before administration/g	Body weight 14 days after administration/g	Weight gain/g	*p* value
^18^F-CB86	432.8 ± 6.85	453.2 ± 8.42	20.3 ± 1.75	435.8 ± 8.75	458.0 ± 5.82	22.6 ± 5.20	0.27

## Data Availability

The data is contained within the article and the Supplementary Materials.

## Supplementary Materials

The following supporting information can be downloaded at: https://www.mdpi.com/article/10.3390/ph19101553/s1, Figure S1: ^1^H-NMR spectrum of compound A1 (400 MHZ, CDCl_3_); Figure S2: ^1^H-NMR spectrum of compound A2 (400 MHZ, CDCl_3_); Figure S3:^1^H-NMR spectrum of compound A3 (400 MHZ, CDCl_3_); Figure S4: ^1^H-NMR spectrum of compound A4 (400 MHZ, CD_3_OD SPE); Figure S5: ^1^H-NMR spectrum of compound A5 (400 MHZ, CDCl_3_); Figure S6: ^1^H-NMR spectrum of compound A7 (400 MHZ, CDCl_3_); Figure S7: ESI-MS spectrum of compound A7; Figure S8: ^1^H-NMR spectrum of compound A8 (400 MHZ, CDCl_3_); Figure S9: ESI-MS spectrum of compound A8; Figure S10: Pre-HPLC of ^18^F-CB86; Figure S11: Purity of ^18^F-CB86 by Radio-TLC at 0 h; Figure S12: Purity of ^18^F-CB86 by Radio-TLC at 2 h; Figure S13: Purity of ^18^F-CB86 by Radio-TLC at 4 h; Figure S14: Purity of ^18^F-CB86 by Radio-TLC at 6 h; Figure S15: PET images overlaid on the MRI image template of the PMOD 4.304 fusion pattern in the whole body and MIP image of the brain; Figure S16: Time–activity curves in the brain after i.v. injection; Table S1: Lipid–water partition coefficient of ^18^F-CB86; Table S2: Biodistribution of radioactivity after i.v. injection of ^18^F-CB86 in Wistar rats (n = 3); Table S3: Body weight changes in rats injected with ^18^F-CB86 or saline (n = 6).
