# Supplementary material for: Synthesis and Preclinical Evaluation of 18F-CB86, a Novel Imidazole[1,2-a] Pyridine-Based TSPO Tracer
Source: Pharmaceuticals (Basel). 2026 Oct 1;19(10):1553. doi: 10.3390/ph19101553 (PMC13708200; doi:10.3390/ph19101553)
Supplement: Supplementary file 1 [file pharmaceuticals-19-01553-s001.zip › pharmaceuticals-4504093-supplementary.pdf]

Article

Synthesis and Preclinical Evaluation of ^{18}F-CB86, a Novel Imidazole[1,2-a] pyridine-Based TSPO Tracer

Fan Zhang [1], Zhanhua Sun [1], Qiang Zheng [1], Zhuoxiao Wang [1], Xilong Tao [1], Baoqi Huo[1], Jing Yu [1,*], Shisheng Wang [2,*]

1 Second Affiliated Hospital, Dalian Medical University, Dalian 116027, China;

2 School of Pharmaceutical Science and Technology, State Key Laboratory of Fine Chemicals, Dalian University of Technology, Dalian 116024, China;

* Correspondence: yujing_2020@dmu.edu.cn (J.Y.); wangss@dlut.edu.cn (S.W); Tel.: +86-411-84694244 (J.Y.).

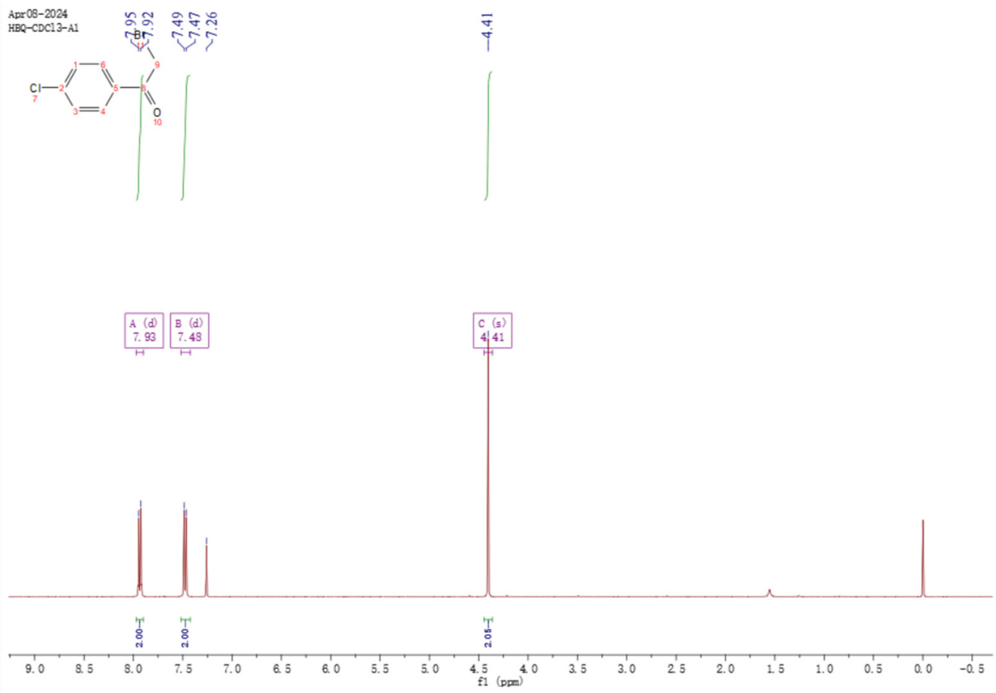

Figure S1. ^{1}H-NMR spectrum of compound A1 (400MHZ, $CDCl_3$)

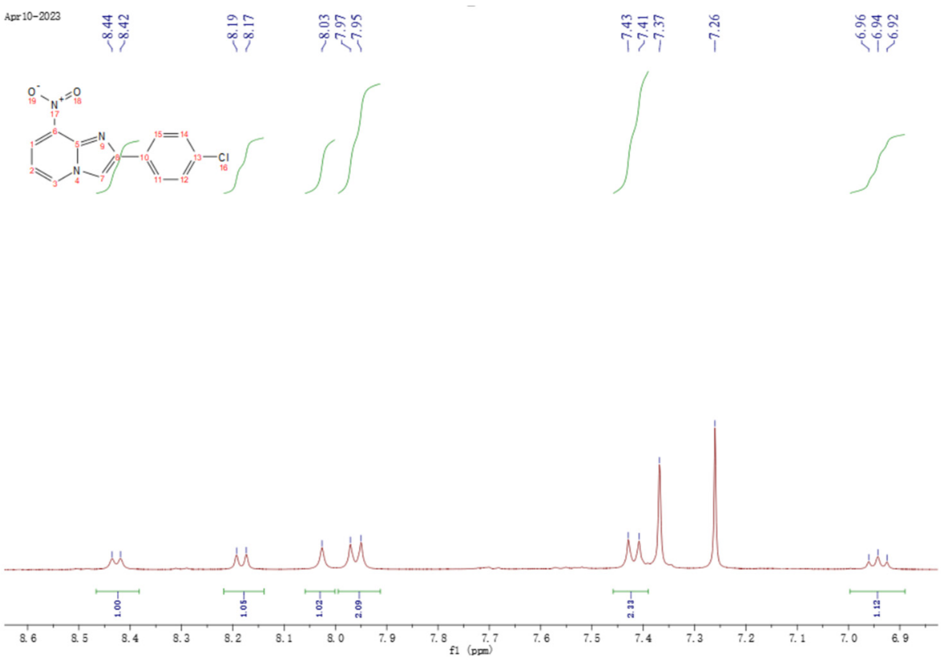

Figure S2. ^{1}H-NMR spectrum of compound A2 (400MHZ, $CDCl_3$)

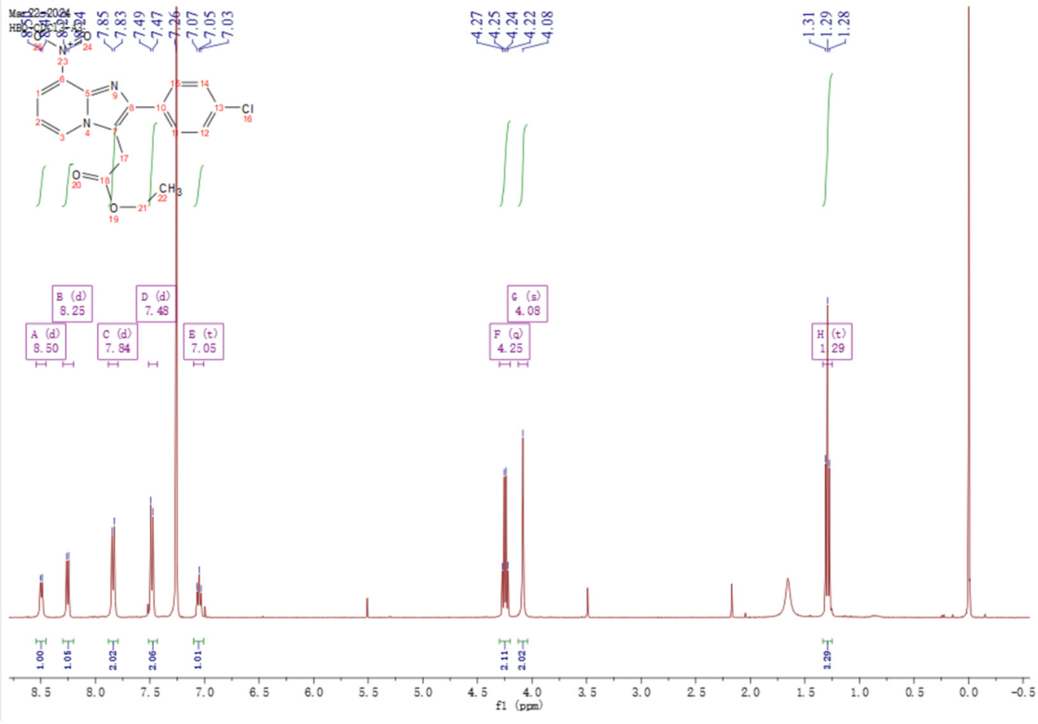

Figure S3. ^{1}H-NMR spectrum of compound A3 (400MHZ, $CDCl_3$)

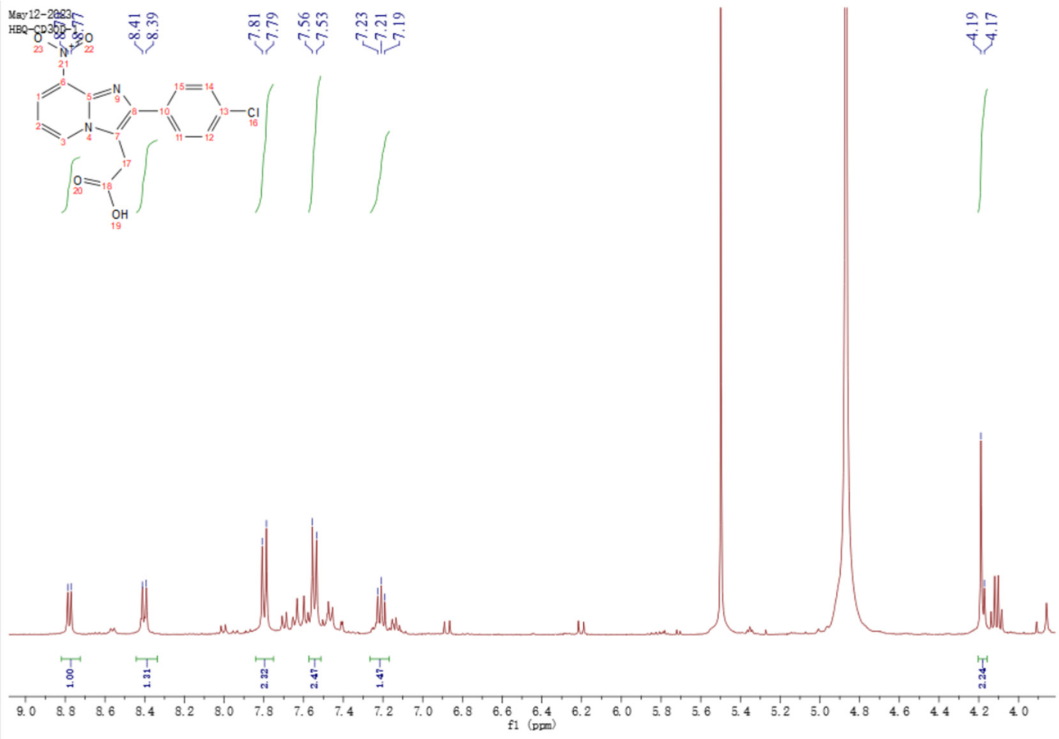

Figure S4. ^{1}H-NMR spectrum of compound A4 (400MHZ, CD_3OD SPE)

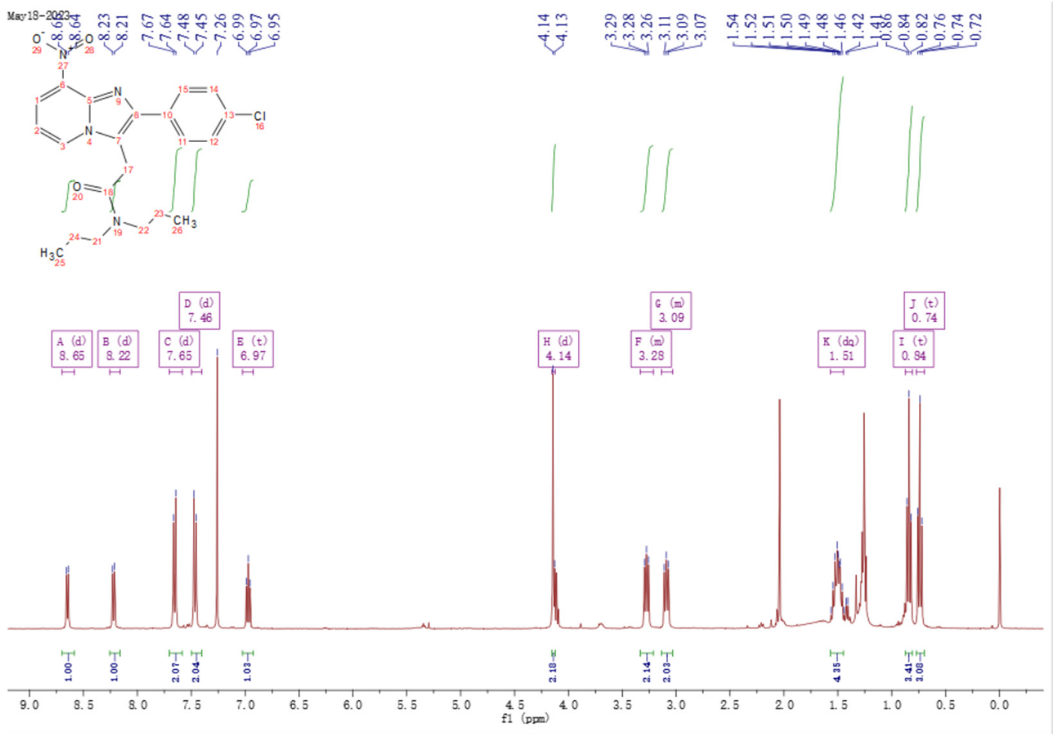

Figure S5. ^{1}H-NMR spectrum of compound A5 (400MHZ, $CDCl_3$)

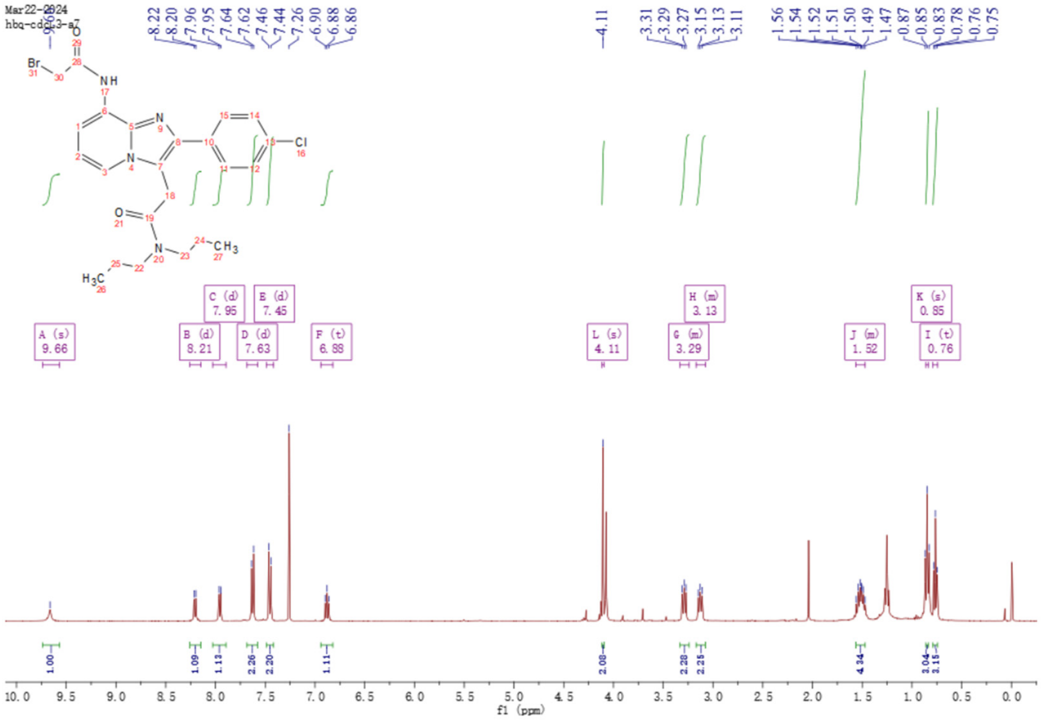

Figure S6. ^{1}H-NMR spectrum of compound A7 (400MHZ, $CDCl_3$)

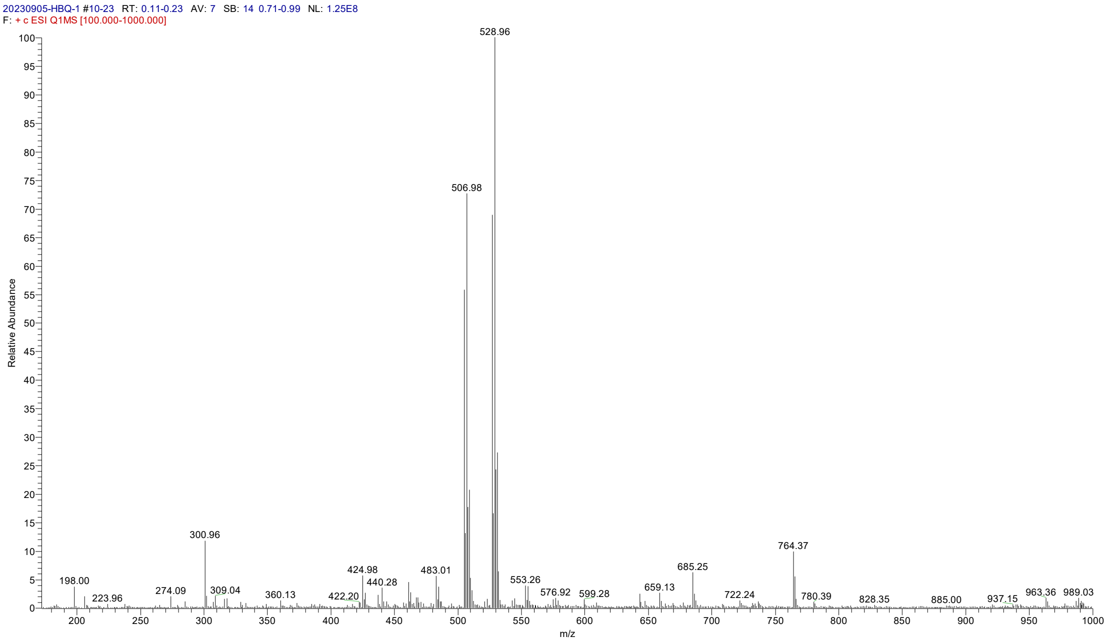

Figure S7. ESI-MS spectrum of compound A7

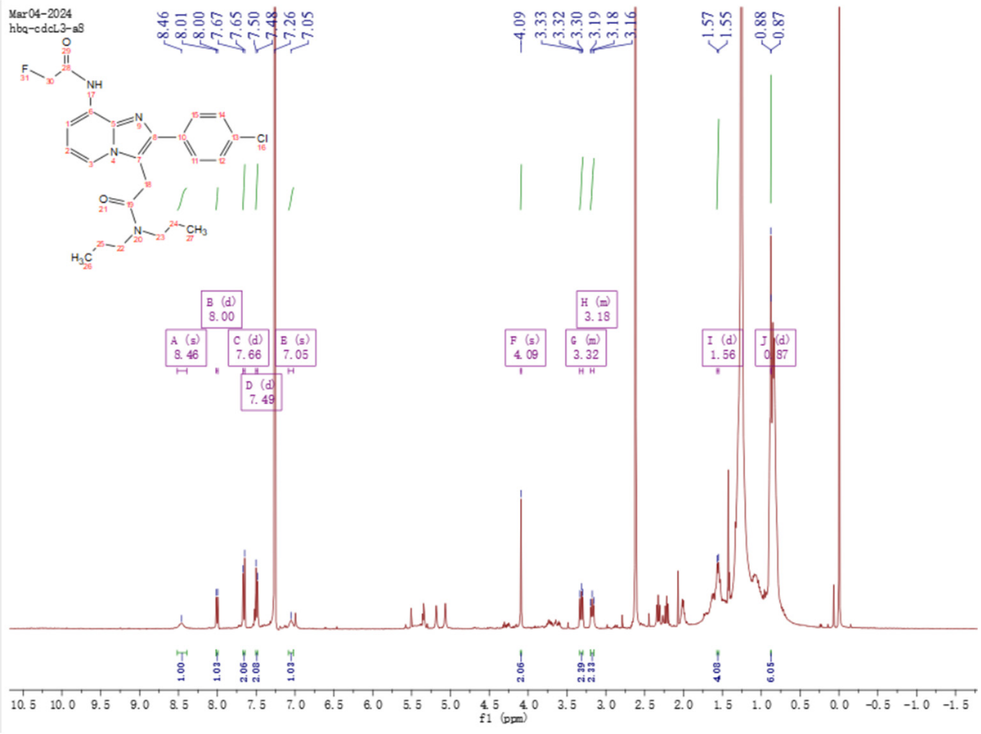

Figure S8. ^{1}H-NMR spectrum of compound A8 (400MHZ, $CDCl_3$)

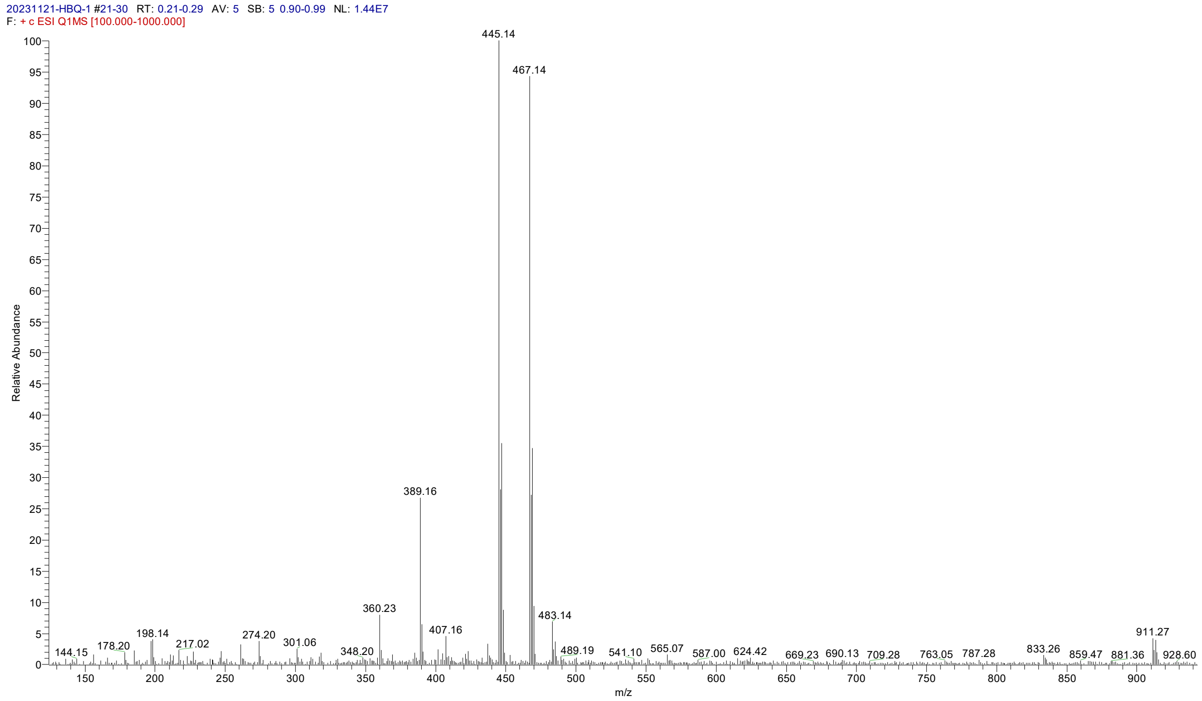

Figure S9. ESI-MS spectrum of compound A8

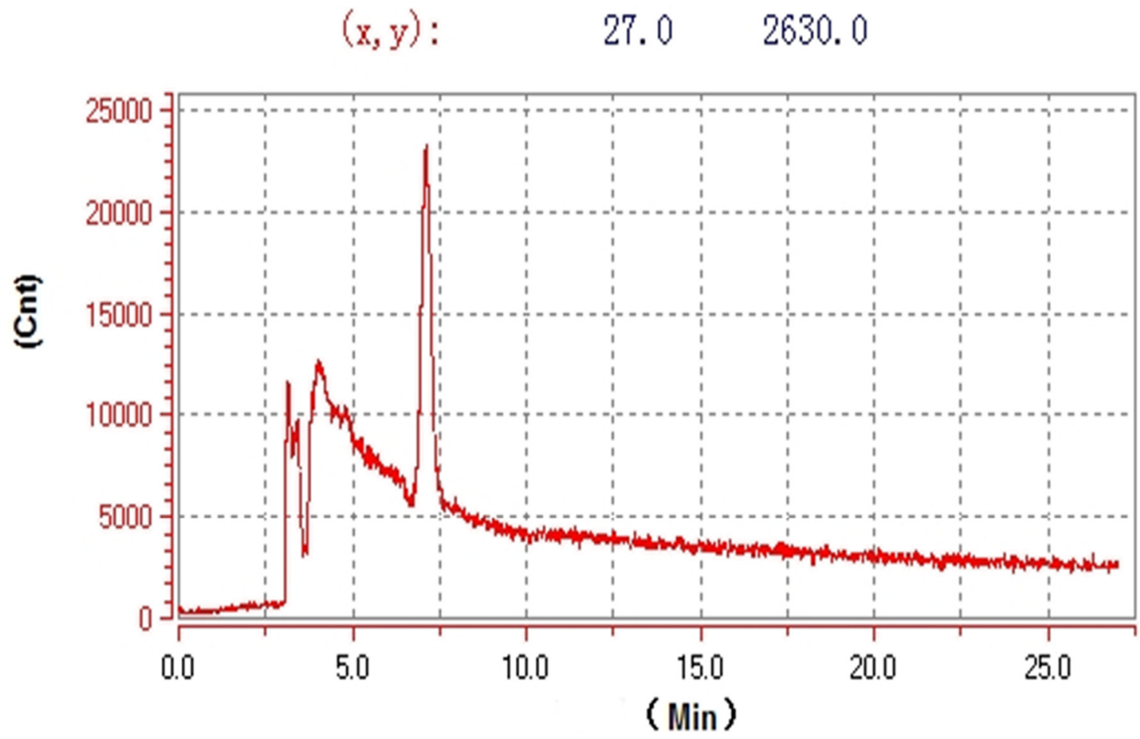

Figure S10. Pre-HPLC of ^{18}F-CB86

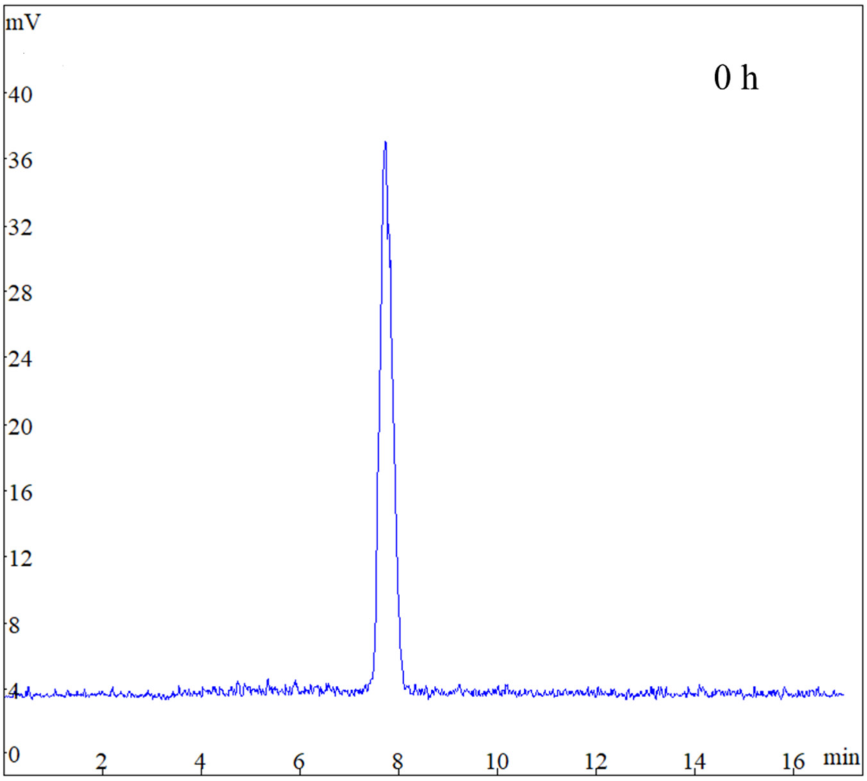

Figure S11. Purity of ^{18}F-CB86 by Radio-TLC at 0 h

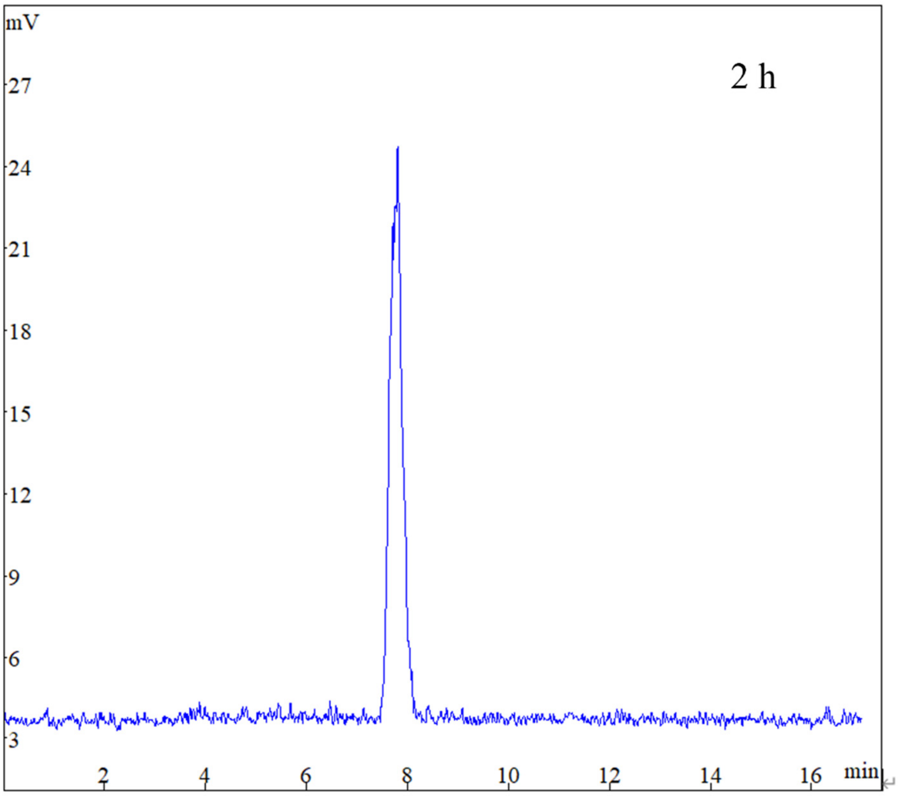

Figure S12. Purity of ^{18}F-CB86 by Radio-TLC at 2 h

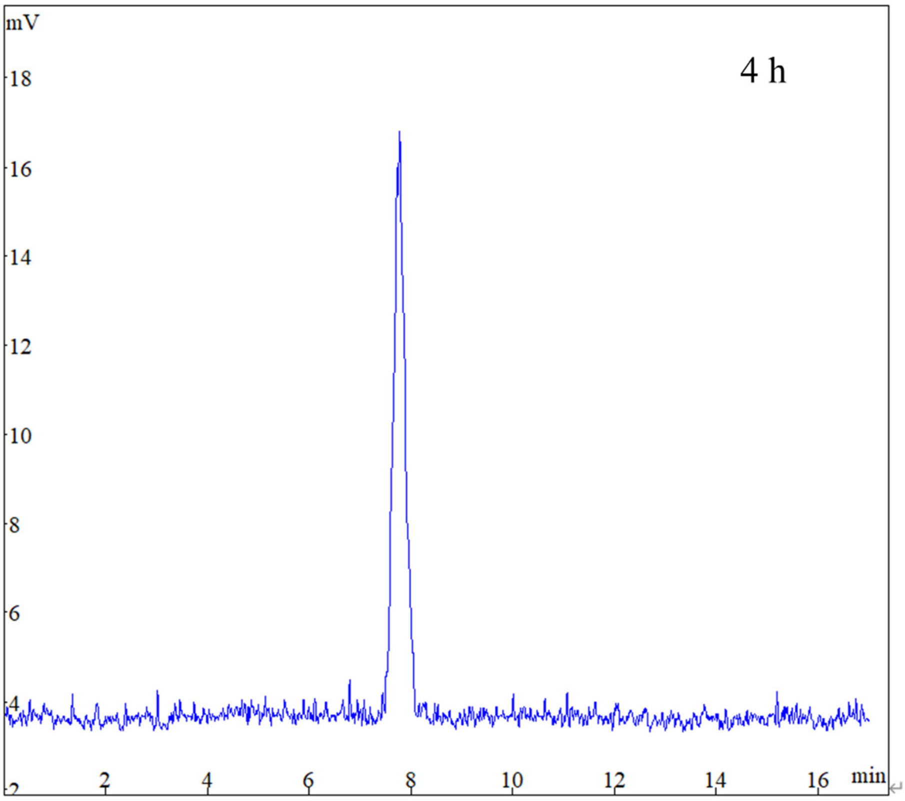

Figure S13. Purity of ^{18}F-CB86 by Radio-TLC at 4 h

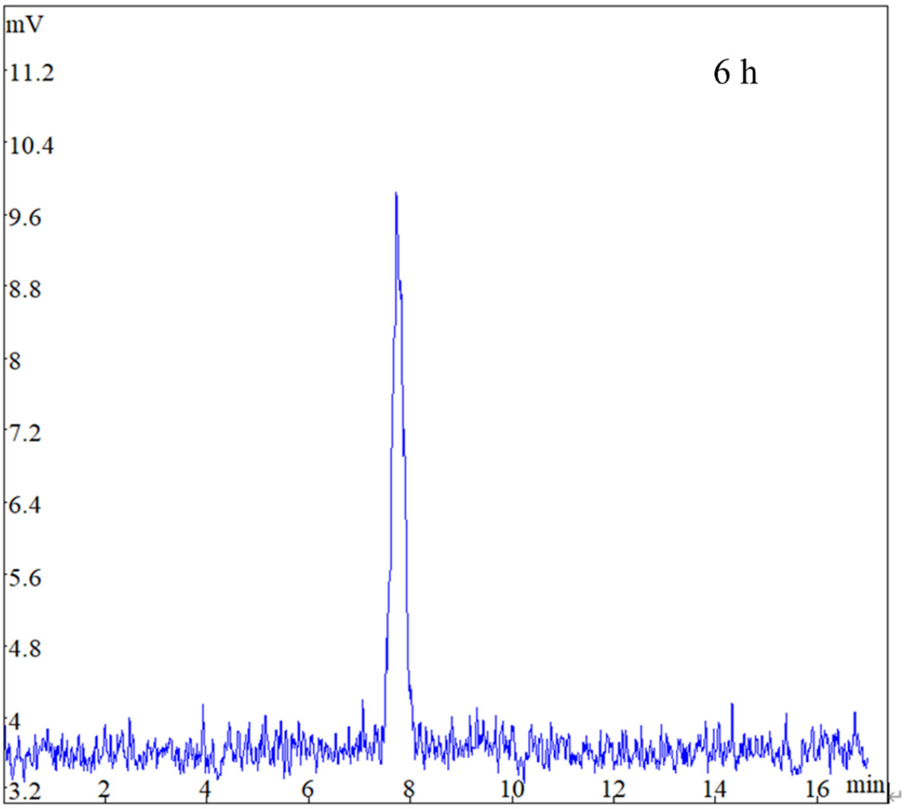

Figure S14. Purity of ^{18}F-CB86 by Radio-TLC at 6 h

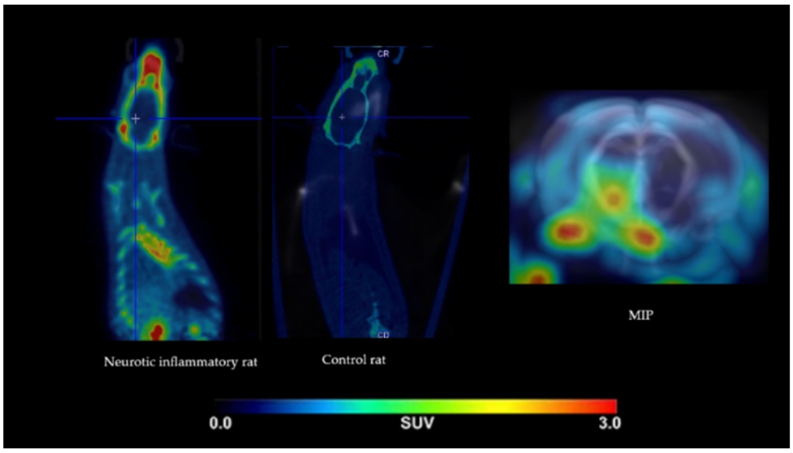

Figure S15. PET images overlaid on the MRI image template of the PMOD 4.304 fusion pattern in the whole body and MIP image of the brain

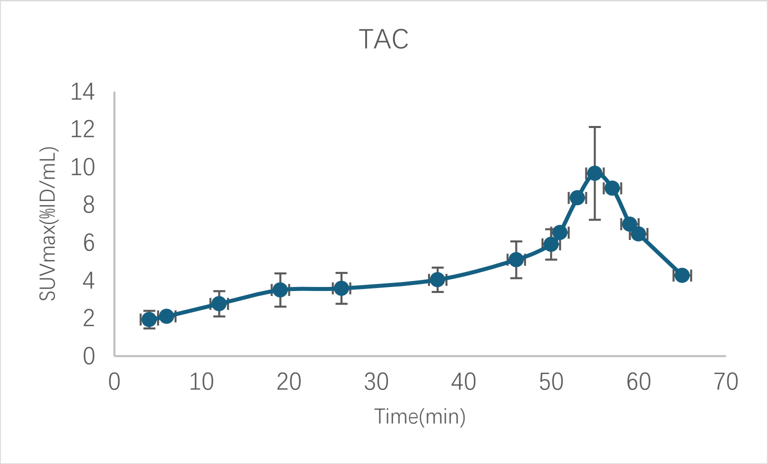

Figure S16. Time-activity curves in the brain after i.v. injection

Table S1. Lipid-Water partition coefficient of ^{18}F-CB86

**number**	**1**	**1**	**1**	**2**	**2**	**2**	**3**	**3**	**3**
**Nonaethylene layer/cps**	9165	8655	9977	8888	9395	10121	9021	9218	9507
**PBS layer/cps**	27321	27410	27604	27580	27428	27184	26704	25274	26931

Table S2. Biodistribution of radioactivity after i.v. injection of ^{18}F-CB86 in Wistar rats (n=3)

Organ	30min	60min	90min	120min
Brain	0.031±0.0031	0.0545±0.0024	0.0511±0.0034	0.0376±0.0069
Heart	0.1706±0.0048	0.1915±0.0131	0.1679±0.0090	0.1565±0.0141
Liver	0.0462±0.0009	0.1594±0.0024	0.1193±0.0117	0.0879±0.0042
Spleen	0.1758±0.0033	0.2023±0.0125	0.1281±0.0143	0.0517±0.0093

Lungs	0.2494±0.0040	0.2000±0.0102	0.2048±0.0248	0.1730±0.0105
Kidneys	0.1793±0.0069	0.3689±0.0143	0.2196±0.0225	0.0937±0.0044
Stomach	0.0299±0.0058	0.0744±0.0076	0.0662±0.0113	0.0582±0.0062
Intestines	0.3963±0.0314	0.1426±0.0126	0.1243±0.0160	0.1335±0.0091
Pancreas	0.0465±0.0007	0.0840±0.0056	0.0539±0.0085	0.0963±0.0134
Muscle	0.0388±0.0014	0.0798±0.0112	0.1015±0.0203	0.0902±0.0135

Table S3. Body weight changes in rats injected with ^{18}F-CB86 or saline (n=6)

	Experimental group			Control Group		
Radiopharmaceutical	Body weight before administration/g	Body weight 14 days after administration/g	Weight gain/g	Body weight before administration/g	Body weight 14 days after administration /g	Weight gain/g
^{18}F-CB86	434	456	22	426	454	28
	421	438	17	444	463	19
	436	457	21	428	452	24
	440	461	21	431	460	29
	429	449	20	447	467	20
	437	458	21	439	455	16
